# Development of a fluorescent quantitative real-time polymerase chain reaction assay for the detection of Goose parvovirus in vivo

**DOI:** 10.1186/1743-422X-6-142

**Published:** 2009-09-15

**Authors:** Jin-Long Yang, An-Chun Cheng, Ming-Shu Wang, Kang-Cheng Pan, Min Li, Yu-Fei Guo, Chuan-Feng Li, De-Kang Zhu, Xiao-Yue Chen

**Affiliations:** 1 Chongqing Academy of Animal Science, Chongqing 402460, Chongqing, China; 2 Avian Diseases Research Center, College of Veterinary Medicine of Sichuan Agricultural University, Yaan 625014, Sichuan, China; 3 Key Laboratory of Animal Diseases and Human Health of Sichuan Province, Yaan 625014, Sichuan Province, China

## Abstract

**Background:**

Goose parvovirus (GPV) is a *Dependovirus *associated with latent infection and mortality in geese. Currently, it severely affects geese production worldwide. The objective of this study was to develop a fluorescent quantitative real-time polymerase chain reaction (PCR) (FQ-PCR) assay for fast and accurate quantification of GPV DNA in infected goslings, which can aid in the understanding of the regular distribution pattern and the nosogenesis of GPV in vivo.

**Results:**

The detection limit of the assay was 2.8 × 10^1 ^standard DNA copies, with a sensitivity of 3 logs higher than that of the conventional gel-based PCR assay targeting the same gene. The real-time PCR was reproducible, as shown by satisfactory low intraassay and interassay coefficients of variation.

**Conclusion:**

The high sensitivity, specificity, simplicity, and reproducibility of the GPV fluorogenic PCR assay, combined with a high throughput, make this method suitable for a broad spectrum of GPV etiology-related applications.

## Background

Goose parvovirus (GPV) is the causative agent of Gosling plague (GP), an acute, contagious, and fatal disease, which is also known as Derzsy's disease [1]. GPV has been formally classified as a member of the genus *Dependovirus *in family *Parvoviridae *[2]. It was first described as a clinical entity by Fang [3]. It causes considerable economic losses, especially in countries with an industrialized goose production system, because the virus infection spreads rapidly worldwide causing high rate of morbidity and mortality [1,4-6].

Regular methods for identifying GPV include agar-gel diffusion precipitin test, virus neutralization (VN) assay, and enzyme-linked immunosorbent assay (ELISA) [5]. However, these methods have certain limitations; they are tedious and are not always reliable because of the requirement of specific-pathogen-free (SPF) gosling embryos and standard positive anti-GPV serum [7,8].

Recently, the highly conserved VP3 region of the GPV gene was cloned and sequenced and analyzed by qualitative polymerase chain reaction (PCR) assays [9-12]. Although qualitative PCR was useful for the diagnosis of GPV infection, it had some problems: it involved the electrophoresis and staining processes, which made the procedure lengthy, increased the risk of contamination, or rendered the method unsuitable for large-scale investigations [13-15]. Moreover, determination of the amount of virus in different tissues and cells was very useful for investigating the nosogenesis, virus replication, host-virus interactions, tropism, and effective for screening anti-viral drugs; all these factors could not be assessed by qualitative PCR [16,17].

In recent years, a method based on PCR with an automatic confirmation phase has been developed. This method, which is known as the fluorescent quantitative real-time PCR (FQ-PCR), has been used widely to quantify the number of genomic copies of pathogenic microorganisms [18,19].

GPV detection by real-time PCR has only been reported by Bi [20]; in that study, the method was not optimized and a FQ-PCR standard curve was not generated. In this study, we reported the optimization of a FQ-PCR assay to quantify GPV DNA in vivo after experimental infection. The results of this study provide some interesting data that may be beneficial to understand the regular distribution pattern and nosogenesis of GPV in vivo in goslings.

## Results

### Concentration of standard pVP3 plasmid DNA

The concentration of standard pVP3 plasmid DNA was 2 μg/μL, and the A260/A280 (ratio) was 1.84; the copy numbers of pVP3 plasmid DNA were 2.76 × 10^11 ^copies/μL.

### Development and optimization of FQ-PCR and conventional PCR

After the optimization of FQ-PCR, we selected the final concentrations of each primer as 0.2 μmol/L and that of probe as 0.16 μmol/L. The MgCl_2 _concentration was adjusted to 10 mM to obtain optimal FQ-PCR assay conditions. Therefore, the optimized 25-μL FQ-PCR reaction system for GPV detection was as follows: 1× PCR buffer, 10 mmol/L MgCl_2_, 0.2 mmol/L dNTPs, 0.2 μmol/L of each primer, 0.16 μmol/L of probe, 1 U Taq, and 1 μL DNA template.

The optimized conventional PCR reaction system used in this study was as described by Huang et al. [12]: 1× PCR buffer, 1.5 mmol/L MgCl_2_, 0.2 mmol/L dNTPs, 1.0 pmol/L of each primer, 2.5 U Taq, and 1 μL DNA template. The optimized annealing temperature was 52°C.

### Establishment of FQ -PCR standard curve

**Figure 1:**
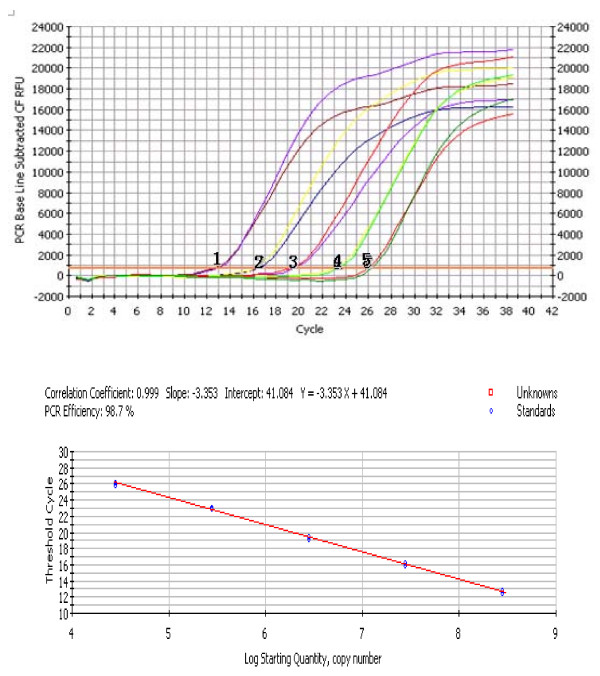
**Establishment of the fluorescent quantitative real-time PCR (FQ-PCR) standard curve**. Ten-fold dilutions of standard DNA ranging from 2.8 × 10^8 ^to 2.8 × 10^4 ^copies/μL were used, as indicated on the x-axis, whereas the corresponding cycle threshold (Ct) values are presented on the y-axis. Each dot represents the result of triplicate amplifications of each dilution. The correlation coefficient and slope value of the regression curve were calculated and are indicated. (1:2.8 × 10^8^, Ct = 12.7; 2: 2.8 × 10^7^, Ct = 16.2; 3: 2.8 × 10^6^, Ct = 19.4; 4: 2.8 × 10^5^, Ct = 22.9; 5: 2.8 × 10^4^, Ct = 25.9)

The FQ-PCR amplification curves and the corresponding FQ-PCR standard curve (Figure 1) were generated by employing the successively diluted known copy numbers of pVP3 for real-time PCR reaction under the optimized conditions. On the basis of the results of correlation coefficient (0.999) and PCR efficiency (98.7%), it was confirmed that the standard curve and the established FQ-PCR protocol were extremely effective. By using the following formula, we were able to quantify the amount of unknown samples: Y = -3.353X + 51.142 (Y = threshold cycle, X = log starting quantity).

### Sensitivity, specificity, reproducibility and dynamic range analysis of the established FQ-PCR

Ten-fold dilutions of the pVP3 plasmid DNA were tested by the established FQ-PCR assay to evaluate the sensitivity of the system, and the detection limit was found to be 2.8 × 10^1 ^copies/reaction. Comparisons were made between the conventional PCR method and our established FQ-PCR method using dilution series of pVP3 plasmid DNA to calculate the end-point sensitivity of each assay. The results indicated that the established FQ-PCR is approximately 1000-times more sensitive than the conventional PCR method; the former method can detect pVP3 copies down to dilutions of 2.8 × 10^1 ^copies/reaction and the latter one that can detect copies up to the dilutions of 2.8 × 10^4 ^copies/reaction.

**Figure 2:**
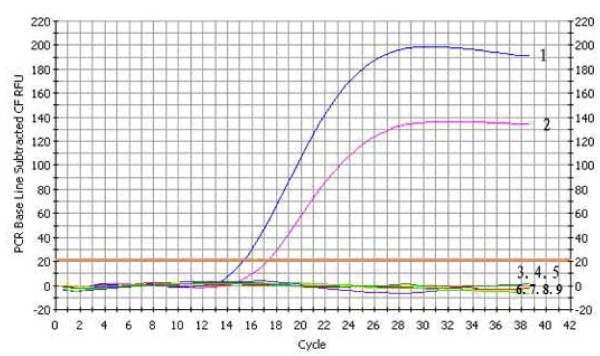
**The specificity of FQ-PCR**. 1. pVP3; 2. GPV-CHv; 3. Aleutian disease virus (ADV); 4. Canine Parvovirus (CPV); 5. Porcine parvovirus (PPV); 6. Newcastle disease viruses (NDV); 7. *Pasteurella multocida *(5:A); 8. *Salmonella enteritidis *(No. 50338); 9. *Escherichia coli *(O78)

The test was performed using DNA from pVP3, GPV-CHv and several other bacteria and viruses as templates to examine its specificity; the result of this analysis showed that none of the bacteria or viruses (other than GPV-CHv and pVP3) yielded any amplification signal, suggesting that the established FQ-PCR assay was highly specific (Figure 2).

The intraassay and interassay CV of this established FQ-PCR was in the range of 0.8-3% for most of the dynamic range (from 2.8 × 10^11 ^to 2.8 × 10^1 ^pVP3 plasmid copies/μL). The results demonstrated that the established FQ-PCR method was characterized by a wide dynamic range (11 logarithmic decades) of detection from 2.8 × 10^11 ^to 2.8 × 10^1 ^pVP3 plasmid copies/μL with high precision. Therefore the dynamic range of the method was between 2.8 × 10^11 ^to 2.8 × 10^1 ^pVP3 plasmid copies/μL, which is relatively broad.

### Dynamic distribution of in vivo GPV test by using the established FQ-PCR assay

Viral load quantification using the established FQ-PCR demonstrated that the GPV DNA copy number of each sample could be calculated using the cycle threshold (Ct) value determined from the standard curve. The dynamic distribution of GPV within the tissues after oral infection with GPV was intermittently determined by means of the FQ-PCR in separate segments of tissues over a 9-day period. Results of this analysis revealed that the blood, heart, liver, spleen, kidney, Bursa of Fabricius (BF), thymus, and Harder's glands were positive at 4-h postinoculation (PI), with about 10^4.93^-10^7.57 ^copies/g. GPV was consistently detected in all the segments of the organs at 8-h PI. The copy numbers of GPV in each tissue reached a peak at 48-72-h PI. Numbers of GPV DNA decreased at 6 days, and by 9 days, the level of GPV DNA decreased remarkably. Importantly, the level of GPV DNA was comparable to that in the other organs at 3-days PI; the liver, spleen, thymus, Harder's glands, and BF had significantly higher numbers of GPV DNA than the rest of the tissues, with >10^10 ^copies/g in the former tissues compared to <10^8 ^copies/g in the rest of the tissues. In addition, the control group did not show any positive results at any time point or in any tissue (Table 1)

**Table 1:** The distribution and quantity of GPV^A ^at different time points^B ^within the different segments of the tissue samples after the goslings were experimentally infected with GPV

**sample**	**4 hr**	**8 hr**	**12 hr**	**24 hr**	**2 days**	**3 days**	**6 days**	**9 days**
Blood	4.93 ± 0.11	5.71 ± 0.10	6.35 ± 0.04	6.81 ± 0.21	7.76 ± 0.10	6.78 ± 0.09	6.51 ± 0.14	4.50 ± 0.23
Heart	5.07 ± 0.04	5.20 ± 0.07	6.18 ± 0.01	7.17 ± 0.07	8.32 ± 0.06	9.07 ± 0.33	8.18 ± 0.05	6.78 ± 0.11
Liver	6.87 ± 0.09	7.66 ± 0.08	8.63 ± 0.17	9.21 ± 0.07	10.39 ± 0.08	11.08 ± 0.10	9.96 ± 0.21	8.08 ± 0.23
Spleen	7.45 ± 0.06	8.71 ± 0.10	9.17 ± 0.07	10.20 ± 0.12	11.16 ± 0.14	11.99 ± 0.07	10.14 ± 0.23	8.97 ± 0.19
Lung	0	5.90 ± 0.19	6.11 ± 0.14	7.75 ± 0.11	7.94 ± 0.21	7.51 ± 0.14	6.00 ± 0.16	4.97 ± 0.02
Kidney	6.98 ± 0.08	7.86 ± 0.11	8.27 ± 0.07	9.15 ± 0.16	9.94 ± 0.14	10.87 ± 0.05	9.34 ± 0.19	7.56 ± 0.16
BF^C^	7.57 ± 0.09	8.25 ± 0.16	8.42 ± 0.14	9.07 ± 0.07	9.85 ± 0.14	10.95 ± 0.14	9.68 ± 0.18	8.84 ± 0.05
Thymus	7.12 ± 0.03	8.27 ± 0.19	8.94 ± 0.13	9.76 ± 0.18	10.39 ± 0.21	11.10 ± 0.07	9.97 ± 0.09	7.97 ± 0.12
Esophagus	0	6.35 ± 0.13	7.97 ± 0.19	8.31 ± 0.16	9.77 ± 0.15	8.48 ± 0.14	8.04 ± 0.14	7.85 ± 0.19
Trachea	0	6.24 ± 0.05	7.61 ± 0.19	8.03 ± 0.05	8.95 ± 0.19	8.11 ± 0.07	6.74 ± 0.18	6.21 ± 0.21
Brain	0	6.62 ± 0.07	7.88 ± 0.05	8.18 ± 0.23	8.97 ± 0.05	9.28 ± 0.21	8.84 ± 0.18	8.27 ± 0.09
HG^D^	7.07 ± 0.16	8.41 ± 0.13	8.96 ± 0.16	9.58 ± 0.16	10.69 ± 0.05	11.20 ± 0.21	10.20 ± 0.18	10.11 ± 0.16
Duodenum	0	7.35 ± 0.18	8.27 ± 0.14	8.37 ± 0.18	8.85 ± 0.09	9.56 ± 0.21	8.72 ± 0.23	7.90 ± 0.23
Jejunum	0	7.29 ± 0.12	7.56 ± 0.21	7.74 ± 0.21	7.83 ± 0.07	8.88 ± 0.15	8.16 ± 0.14	7.64 ± 0.21
Ileum	0	7.76 ± 0.18	7.90 ± 0.18	8.18 ± 0.23	8.78 ± 0.14	9.45 ± 0.21	8.61 ± 0.23	7.87 ± 0.15
Cecum	0	6.41 ± 0.12	6.86 ± 0.14	7.10 ± 0.21	7.43 ± 0.05	8.04 ± 0.12	7.10 ± 0.21	6.87 ± 0.09
Rectum	0	6.17 ± 0.16	6.33 ± 0.12	6.71 ± 0.19	7.28 ± 0.12	7.95 ± 0.19	7.45 ± 0.16	6.67 ± 0.21

A GPV = Goose parvovirus

B Units: log10 copies/ml for blood and log10 copies/g for others

C BF = Bursa of Fabricius

D HG = Harder's glands

## Discussion

Here, we describe a real-time PCR assay for the quantification of GPV genome coupes in goslings. We confirmed that this assay was highly sensitive, specific, and reproducible.

Real-time PCR has become a potentially powerful alternative in microbiological diagnostics because of its simplicity, rapidity, reproducibility, and high sensitivity compared to other diagnostic methods [21-23]. In this study, we clearly established the applicability of real-time PCR for the quantification of GPV because of its remarkable sensitivity and high-throughput potential, which is beyond the scope of other diagnostic methods.

The real-time PCR assay permits the simultaneous detection and quantification of DNA. It is useful for understanding the pathogenesis of the disease and the mechanisms of virus transmission by enabling the investigation of viral dynamics [21]. The assay can be used to determine the amount of viral DNA in different tissues at various times after infection; this infection data could be interesting and useful for expanding the understanding on viruses. Quantification of the viral load makes it possible to study the kinetics and tropism of GPV in different birds, tissues, and cells. Our study is different from other studies that examined the distribution of viruses and the characteristics of the lesions induced in experimentally infected geese and Muscovy ducts by performing comparative pathological studies or other assays [24,25].

Previous studies have examined the distribution of GPV in infected Muscovy ducks by qualitative PCR [9], including a study that used quantitative PCR [20]. However, Bi et al. did not optimize the FQ-PCR assay for future application. Limn et al. found that GPV could be first detected at 2-d PI in the liver and other organs. Because the real-time PCR method was more sensitive than regular qualitative PCR methods [26], we could first detect GPV at 4-h PI in the liver and other tissues, which was less than 40 h compared to the time required by regular qualitative PCR methods. This finding is important because the prevention and early detection are presently the most logical strategies for virus control [27].

Islam et al. reported that in orally infected ducks, duck plague virus (DPV) first invaded the epithelial cells of the intestinal tract, following which it was transported to other immune organs, such as BF, thymus, and spleen, from where it finally invaded to all the other host tissues via blood circulation [28]. Similarly, our study showed that GPV was distributed in the blood, heart, liver, spleen, kidney, BF, thymus, and Harder's glands at 4-h PI. Subsequently, GPV was consistently distributed in all the segments of the organs at 8-h PI. The copy numbers of GPV in the liver, spleen, thymus, Harder's glands, and BF was significantly higher than that in the other regions. Therefore, these immune organs could be considered as the primary sites of invasion in normal goslings after GPV infection.

Live GPV vaccine is widely used to immunize adult geese to prevent GPV infection [12]. Real-time PCR and qualitative PCR assays [10-12] can amplify the highly conserved VP3 region of the GPV gene, which is distributed in the high-virulence strain and live-vaccine strain of GPV. Theoretically, these methods would not be able to differentiate the GPV vaccine strain from the high-virulence strain; nonetheless, we could perform the study on the dynamic distribution of GPV in vivo using these methods, because the animals were certificated as GPV-free by qualitative PCR assay before being infected with the high-virulence strain. For standardization, the VP3 gene was cloned into a plasmid. The available live vaccine could have been used as the standard.

## Conclusion

In conclusion, the established real-time PCR assay was rapid, sensitive, and specific for the detection and quantification of GPV DNA. In addition, our results provide significant data for clarifying that the immune organs were the primary sites of GPV invasion in infected goslings.

## Methods

### Virus and PCR template DNA preparation

GPV CH_V _strain, a high-virulence strain of GPV, was obtained from Key Laboratory of Animal Diseases and Human Health of Sichuan Province.

Aleutian disease virus (ADV), canine parvovirus (CPV), porcine parvovirus, (PPV), Newcastle disease virus (NDV), *Pasteurella multocida *(5: A), *Salmonella enteritidis *(No. 50338), and *Escherichia coli *(O78) were provided by Key Laboratory of Animal Diseases and Human Health of Sichuan Province.

Template DNA was extracted from the viral and bacterial stock solutions using the High Pure PCR Template Preparation kit (Roche Diagnostics GmbH, Mannheim, Germany) according to the manufacturer's instructions.

### PCR primer and probe design

The FQ-PCR assay primers and probe (namely, GPV-F, GPV-R, and CPV-FP) were designed on the basis of the highly conserved VP3 region of GPV (GenBank Accession No. U25749). Primers and probe were designed by using the Primer Premier software (version 5.0). The position and sequence of the primers and probe are shown in Table 2. The product size was 60 bp. The fluorogenic probe was labeled at the 5' position with 6-carboxyfluorescein (FAM) dye as a reporter and at the 3' position with tetra-methylcarboxyrhodamine (TAMRA) as a quencher and with Minor Groove Binder (MGB™).

**Table 2:** Oligonucleotide sequences of the primers and probes used in the GPV FQ-PCR method (Oligonucleotide positions have been determined by referring to the gene sequence of U25749)

**Name**	**Sequence 5' to3'**	**Position**	**Amplicon size (bp)**
GPV-F	GTGCCGATGGAGTGGGTAAT	3084-3103	60
GPV-R	ACTGTGTTTCCCATCCATTGG	3122-3143	
GPV-FP	6FAM-FTCGCAATGCCA ATTTCCCGAGGP--TAMRA	3098-3120	
VP3-1	AAGCTTTGAAATGGCAGAGGGAGGA	3008-3033	1658
VP3-2	GGATCCCGCCAGGAAGTGCTTTATTTGA	4637-4665	

The sequences of the forward and reverse primers used for the conventional PCR were as described by Huang et al., and this primer pair yielded a 441-bp amplicon [12].

All the probes and primers were synthesized by TakaRa Biotech Co., Ltd. (Dalian, China) and purified by the corresponding high-performance liquid chromatography (HPLC) system.

### Preparation of standard plasmid DNA templates

The recombinant plasmid DNA (namely, pVP3) and primer constructs (namely, VP3-1 and VP3-2) were designed to amplify an expected 1658-bp PCR product that included positions 3,008-4,665 bp of GPV (GenBank Accession No. U25749) (Table 2). Primers were designed by using the Primer Premier software (version 5.0). The product was ligated into the pGM-T vector (Tiangen Corp., Beijing, China) and transformed into *E. coli *DH5α competent cells [27]. The pVP3 was extracted using the TIANprep plasmid extraction kit (Tiangen Corp., Beijing, China). The pVP3 DNA concentration was determined by measuring the absorbance at 260 nm using a Smartspec 3000 spectrophotometer (Bio-Rad Corp., Hercules, CA), and the purity was confirmed using the 260/280 nm ratio. On the basis of the molecular weight, we calculated the pVP3 copy number using the equations described by Ke [29].

### Development and optimization of FQ-PCR

The FQ-PCR was performed using the ABI AmpliTaq Gold DNA polymerase system with an icycler IQ Real-time PCR Detection System (Bio-Rad Corp., Hercules, CA) according to the manufacturer's instructions. The reaction, data acquisition, and analysis were performed using iCycler IQ optical system software. The FQ-PCR was performed in a 25-μL reaction mixture containing 1× PCR buffer, 0.3 mmol/L dNTPs, 1.25 U Taq, and 1 μL DNA template according to the manufacturer's instructions. Autoclaved nanopure water was added to make the final volume to 25 μL. Each run comprised an initial activation step of 30 s at 95°C, followed by 40 cycles of denaturation at 94°C for 10 s and annealing at 60°C for 30 s; the fluorescence was measured at the end of the annealing/extension step. The tests were performed using 0.2-mL PCR tubes (ABgene, UK). FQ-PCR reactions were optimized in triplicate based on the primer, probe, and MgCl_2 _concentration selection criteria, which was performed according to 4 × 4 × 4 matrix of primer concentrations (0.10, 0.12, 0.16, and 0.20 μmol/L), probe concentrations (0.10, 0.12, 0.16, and 0.20 μmol/L), and MgCl_2 _concentrations (1.0, 5.0, 10.0, and 15.0 mmol/L). Conditions were selected to ensure that both the fluorescence acquisition curves were robust and Ct values were the lowest possible to the known template DNA concentrations.

An internal positive control was introduced into the FQ-PCR assay to verify that DNA was not lost during the extraction step and PCR inhibitors were absent in the DNA templates as described by Guo et al. [27].

### Establishment of the FQ-PCR standard curve

The FQ-PCR standard curve was generated by successive dilutions of pVP3 with known copy numbers. The purified pVP3 plasmid DNA was serially diluted 10-fold in TE buffer, pH 8.0, from 2.8 × 10^8 ^to 2.8 × 10^4 ^plasmid copies/μL. These dilutions were tested in triplicate and used as quantification standards to construct the standard curve by plotting the plasmid copy number logarithm against the measured Ct values. The Bio-Rad iCycler IQ detection software was used to generate the standard curve and to calculate the correlation coefficient (R2) of the standard curve and the standard deviations of the triplicate samples.

### FQ-PCR sensitivity, specificity, reproducibility, and dynamic range analysis

The sensitivities of the conventional PCR and FQ-PCR were each determined using triplicates of different concentrations of the recombinant plasmid pVP3. Template DNA was prepared as follows: plasmids of pVP3 were serially diluted 10-fold from 2.8 × 10^6 ^copies/μL to 2.8 × 10^0 ^copies/μL using sterile ultra pure water. From each dilution, 1 μL was used as a template and subjected to the conventional PCR and FQ-PCR protocol. The detection limit of the conventional PCR was determined based on the highest dilution that resulted in the presence of clear and distinct amplified fragments (441 bp) on the agarose gel. The detection limit of the FQ-PCR was determined based on the highest dilution that resulted in the presence of Ct value in real-time PCR detection.

DNA from pVP3, GPV-CHv and several other pathogens, including ADV, CPV, PPV, NDV, *Pasteurella multocida *(5: A), *Salmonella enteritidis *(No. 50338), and *Escherichia coli *(O78) (kindly provided by Key Laboratory of Animal Diseases and Human Health of Sichuan Province) were used as templates in the triplicate analyses to confirm the specificity of the technique.

Within-run and between-run reproducibilities of the FQ-PCR assay were assessed by multiple measurements of pVP3 samples of different concentrations. The assay was conducted by assessing the agreement between the replicates in five replicates (within-run precision) and in five separate experiments (between-run precision) of the serially diluted pVP3 plasmid samples through transforming the raw data to their common logarithms and performing analysis of the mean coefficient of variation (CV) values of each pVP3 standard dilution [27].

Dilutions of pVP3 plasmid were used to determine the dynamic ranges of the FQ-PCR assay. The lower and upper limits of quantification were defined by the pVP3 recombinant standard plasmid sample concentrations possessing reasonable precision [27].

### Goslings and tissue preparation

GPV-free goslings (10-day-old) that were certificated with qualitative PCR as described by Huang [12] were obtained from the breeding facility of the Institute of Poultry Sciences in Sichuan Agricultural University, China. Animals were bred and maintained in an accredited facility at the Institute of Poultry Sciences in Sichuan Agricultural University (Sichuan, China), and the experiments conducted during this study conform to the principles outlined by the Animal Welfare Act and the National Institutes of Health guidelines for the care and use of animals in biomedical research.

Fifty goslings were randomly divided into 2 groups. In brief, a group of 40 goslings were orally infected with GPV CH_V _strain, using 0.1 mL of 10^3 ^LD_50 _per gosling. Another group of 10 goslings was treated with an equal volume of physiologic saline and used as a control [20].

Three goslings from the infected group and 1 gosling from the control group were killed at each time point. Blood, heart, liver, spleen, lung, kidney, BF, thymus, esophagus, trachea, brain, Harder's glands, duodenum, jejunum, ileum, cecum, and rectum were analyzed by the real-time PCR at different postinoculation (PI) time points, at 30 min; 1, 2, 4, 8, 12, and 24 h; and 2, 3, 6, and 9 days. Tissues were surgically removed from the goslings and frozen at -80°C, weighed, and homogenized using an Omni PCR Tissue Homogenizer (Omni). Normal tissue sample sizes were 20 mg. For the assays, tissue samples were homogenized in 1 mL of phosphate buffered saline (PBS, pH 7.4). The homogenizer was washed multiple times between each tissue homogenization. DNA was extracted from the tissue samples by using the method described by Cheng [30]. Using this assay, we could quantify the viral load. All the samples were analyzed 3 times. The viral concentrations were expressed as the mean log_10 _virus genome copy numbers per g or 1 mL of the tested tissue or blood.

## Competing interests

The authors declare that they have no competing interests.

## Authors' contributions

JY carried out most of the experiments and wrote the manuscript. AC and MW critically revised the manuscript and the experiment design. KP, ML, YG, CL, DZ and XC helped with the experiment. All of the authors read and approved the final version of the manuscript.
